# Extraction of Metals from Polluted Soils by Bioleaching in Relation to Environmental Risk Assessment

**DOI:** 10.3390/ma15113973

**Published:** 2022-06-02

**Authors:** Ioana Monica Sur, Valer Micle, Andreea Hegyi, Adrian-Victor Lăzărescu

**Affiliations:** 1 Department of Environment Engineering and Entrepreneurship of Sustainable Development, Faculty of Materials and Environmental Engineering, Technical University of Cluj-Napoca, 103-105 Muncii Avenue, 400641 Cluj-Napoca, Romania; 2 NIRD URBAN-INCERC Cluj-Napoca Branch, 117 Calea Floresti, 400524 Cluj-Napoca, Romania; adrian.lazarescu@incerc-cluj.ro

**Keywords:** 9K medium, bioleaching, *Thiobacillus ferrooxidans*, pollution indices, ecological risk

## Abstract

Environmental pollution has particular implications for the whole geosystem and increases the global risk to human and ecological health. In this regard, investigations were carried out on soil samples to perform the quality status assessment by determining: pH, texture, structure and metal concentration, as well as carrying out an assessment of anthropogenic activity by determining pollution indices: C_f_ (contamination factor), C_d_ (degree of contamination), PLI (pollution load index), E_r_ (ecological risk index) and PERI (potential ecological risk index). Analyses on soil samples showed high concentrations of metals (Cu: 113–2996 mg kg^−1^; Pb: 665–5466 mg kg^−1^; Cr: 40–187 mg kg^−1^; Ni: 221–1708 mg kg^−1^). The metal extraction experiments were carried out by bioleaching using *Thiobacillus* *ferrooxidans*, microorganisms at different amounts of bioleaching solution (20 mL and 40 mL 9K medium) and a stirring time of up to 12 h. The results on the degree of contamination, pollution loading index PLI (2.03–57.23) and potential ecological risk index PERI (165–2298) indicate that the soils in the studied area have a very high degree of pollution. The decontamination procedure by bioleaching showed a decrease, but at the end of the test (12 h), the followed indices indicate high values, suggesting that bioleaching should continue. The depollution yield after 12 h of treatment is, however, encouraging: Cu 29–76%, Pb: 10–32%, Cr: 39–72% and Ni 44–68%. The use of yield–time correlation equations allows the identification of the optimal exposure time on the bioleaching extraction process to obtain optimal results. The aim of the research is to determine the soil quality, soil environmental risk, extraction of metals from polluted soils by bioleaching and to identify influencing factors in achieving high remediation yields.

## 1. Introduction

The interest in a cleaner and healthier environment has increased in recent years, becoming a pressing need due to the awareness of the negative impact that pollution has on the quality of life and health of the population [1,2].

The presence of metals generates the most widespread chemical pollution of soil, and its adverse effects are particularly strong. Metal concentrations in uncontaminated dry soils in Romania range from: Cu 20–40 mg kg^−1^; Pb 22 mg kg^−1^; Cr 10 mg kg^−1^; Ni 25–40 mg kg^−1^ [3]. Naturally high levels of metals can be found in soil as a result of geological processes. However, concentrations are mostly very high due to anthropogenic influences from excessive agriculture and intensive industrial pollution [4,5,6,7].

The accumulation of metals in soil affects the surrounding environment through quantity and toxicity, but also through chemical bonds that influence soil reaction, resulting in degradation or loss of soil functions [6,7,8]. Soil pollution with metals affects the physical and chemical properties of soil. High concentrations of Cu alter the humus composition, and Pb inhibits enzymatic processes, reducing the intensity of carbon dioxide removal, both of them reducing the number of microorganisms and nutrient uptake by plants [6,9,10].

These pollutants also affect the health of the population, because copper compounds, through ingestion, can cause digestive and nervous disorders. In high concentrations these copper compounds can even trigger paralysis or cardio-respiratory arrest. Lead is a metal that can cause, even in very low concentrations, serious damage to the brain and nervous system [4,5,10]. Chromium is deposited in the liver, spleen and kidneys, and nickel is carcinogenic and, in some people, can cause allergies [7,10].

The phenomenon of environmental pollution is no longer local or regional, but global, with particular implications for the entire geosystem and leading to an increase in the global risk to human and ecological health [1,2,4,5].

Bioleaching, or bacterial leaching, has gained increased attention as it is innovative, environmentally friendly, and economical [11,12]. The bioleaching process relies on the ability of micro-organisms to transform solid compounds into soluble and extractable elements that can be recovered [13,14]. Acidophile microorganisms (*Sulfolobus acidocaldarius*, *Acidithiobacillus*, *Leptospirillum* and *Thiobacillus ferrooxidans*) live in highly acidic environments (pH 1–3.0) and in the presence of very high concentrations of metals [14,15,16,17,18].

The microorganisms or bacteria that are used in the aerobic bioleaching process directly or indirectly oxidize inorganic compounds. Oxidation and acid-producing activities by sulfo-oxidizing bacteria are essential [13].

In the direct process, bacteria can directly oxidize the sulfur ion from metal sulfide into sulfate. This process is shown in Equations (1) and (2) [13,19,20]:

(1) $$\mathrm{MS}\to M^{2+}+S^{2-}$$

(2) $$S^{2-}+2O_{2}\overset{\mathrm{bacteria}}{\to }{\mathrm{MSO}}_{4}^{2-}$$

A general reaction is used to express the biological oxidation of a metal sulfide involved in bioleaching. Metal sulfides can be iron-based (pyrites) or other metal-based (Equation (3)) [13]:

(3) $$\mathrm{MS}+2O_{2}\overset{\mathrm{bacteria}}{\to }{\mathrm{MSO}}_{4}$$

In the indirect process, the oxidation of metal sulfides is done by microbially generated ferric ions (Equation (4)):

(4) $$\mathrm{MS}+2{\mathrm{Fe}}^{3+}\to M^{2+}+2{\mathrm{Fe}}^{2+}+S^{0}$$

The ferrous iron that is formed is re-oxidized by bacteria. When sulfur is formed, the presence of bacteria is indispensable for its oxidation to sulfuric acid and thus maintaining the solubilized metal (Equation (5)) [13]:

(5) $$S+H_{2}O+3/2O_{2}\overset{\mathrm{bacteria}}{\to }H_{2}{\mathrm{SO}}_{4}$$

The production of sulfuric acid maintains the acidity of the solution at a favorable pH, and leads to the growth of ferro-oxidizing and sulfo-oxidizing bacteria and the solubilization of metals [14]. The general indirect leaching process involves the ferric–ferric cycle [19] (Equation (6)):

(6) $$4{\mathrm{FeSO}}_{4}+{\;O}_{2}+2H_{2}{\mathrm{SO}}_{4}\overset{\mathrm{bacteria}}{\to }2{\mathrm{Fe}}_{2}{\left({\mathrm{SO}}_{4}\right)}_{3}+2H_{2}O$$

**Figure 1:**
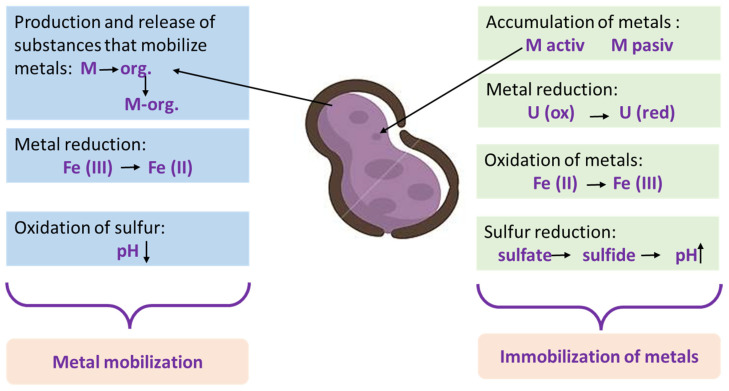
Interactions between metals and microorganisms.

Microorganisms can alter the mobility of metals in the environment through its physical or chemical changes by microbial redox reactions. These microorganisms help in the development of mechanisms that alter solubility, mobility and/or toxicity (Figure 1) and allow soil remediation by separating or dissolving contaminants [21]. Separation or immobilization involves mechanisms of bioabsorption (uptake of contaminants into biomass), as well as changes in the redox state (reduction of oxidized metal to an insoluble form), and the accumulation, precipitation and/or volatilization of pollutants by phytoremediation. Dissolution or mobilization includes the processes of bioleaching metals and metalloids and changes in the redox state (oxidation of small insoluble metal forms to soluble forms), which favor dissolution or volatilization. The removal of inorganic pollutants can be achieved according to the following principles: (1) after precipitation, the pollutants are immobilized; (2) the concentration of the pollutant reduces the volume of the contaminated matrix; (3) the separation of metals is in an environment with lower risk potential [21].

Organisms are exposed to the presence of different types of metals and metalloids in the environment. Interaction between them develops defense mechanisms, which sometimes generates benefits and sometimes is to their detriment [21]. Microorganisms use metals for structural and/or catalytic functions and have the ability to bind metal ions present on the outside of the cell surface or transport them to the cell interior for various intracellular functions (Ca and Mg have structural and catalytic functions, V, Cr, Mn, Fe, Co, Ni, Cu, Zn, Mo and Se in low concentrations can participate in catalytic functions). Some prokaryotic microorganisms can use, during metabolism, metal species that may exist with different valences, such as Cr, Mn, Fe, Co, Cu and Ca, because they act as acceptors or donors of electrons [21].

Having a very rich and mobile enzymatic apparatus, the microorganisms have a true advantage in their use for biotechnological soil depollution processes. This also mainly happens because they have a high biosynthesis capacity and a high adaptability to environmental conditions. Generally, the following microorganism species are used for the bioleaching of metals: *Thiobacillus*, *Leptospirillum* or *Sulfolobus* [10].

*Thiobacillus ferrooxidans*, are single-celled, rod-shaped microorganisms, 1–2 microns long and 0.5–1.0 microns wide. They are tolerant of high acidity and are found in soils with a pH of 2.2, continuing to live even at pH below 0.6, but these organisms grow best at pH 2–5 and at temperatures between 25–35 °C [22].

*Thiobacillus ferrooxidans* use Fe^2+^ as an energy source. Equation (7) shows the oxidation relationship of Fe^2+^:

(7) $$4{\mathrm{Fe}}^{2+}+O_{2}+4H^{+}\to 4{\mathrm{Fe}}^{3+}+2H_{2}O$$

The assessment of soil metal contamination is done by calculating several parameters that indicate the pollution of the soil. These parameters are used to monitor soil quality and ensure future sustainability [23,24] by highlighting the degree of contamination and the potential ecological risk [25,26].

To highlight the influence of anthropogenic activities on soil quality and to assess soil metal pollution, a simultaneous evaluation of the contamination index (C_f_), degree of contamination (C_d_), pollution index (PLI), ecological risk factor (E_r_) and potential ecological risk index (PERI) parameters can be performed [27,28,29].

The PLI index is used for the overall assessment of the degree of contamination in the soil and provides an easy way to demonstrate the deterioration of soil conditions due to metal accumulation [23]. The PERI index allows the identification of various environmental effects (toxicology, environmental chemistry and ecology) and can assess the environmental risks caused by metals [8,30].

Rouchalova (2020) pointed out that particle size (71–100 μm), pH (1.8) and microorganism density (9K medium) are the most important parameters in achieving high depollution yields [31]. Groudev et al. (2001) pointed out that microbial activity is the most important factor in Cu, Zn, Cd and As bioleaching, the exception being Pb [32].

Studies in the literature have shown that bioleaching is efficient for the extraction of metals from water treatment sludge (Cr 92.6%, Cu 80.6%, Fe 95.6%, Mg 91%, Ni 89.7%, Pb 99.5%, Zn 93% [33,34,35], Fe 76.5%, Cu 82%, Pb 89.9%, Zn 90%) [31] or from municipal and industrial waste [36]. Pyrotite bioleaching could increase Fe recovery by bacterial adaptation and biological contact oxidation [37], and Fe can be removed (18%) by adding 9K medium [38].

Results obtained in the literature regarding the bioleaching of metals from soils, obtained under similar experimental conditions (use of *Thiobacillus* microorganisms, sample agitation (120–170 rpm), extraction time (5–48 days) and temperature (28–30 °C)) have shown that good extraction yields can be obtained for: Cu: 44% [39], 46% [40], 20–73% [41], 72. 8% [42], 69–92% [43], 95% [44,45], 51–72% [46], 78% [47], 80.6% [33,34,35], 82% [31], 83% [48], 89–96% [49], 95–96% [50]; Pb: 10–54% [43], 16–60% [50], 18% [40], 33–72% [51], 39.4% [42], 75–84% [49]; Ni: 10–47% [50], 35–65% [51], 69–92% [43], 75–93% [49], 78% [40], 90% [33,34,35]; and Cr: 14% [40], 10–41% [43], 9–20% [51], 53–92% [49], 64% [39].

As a result, based on the information present in the literature, it can be said that in order to obtain a high yield of soil metal extraction technologies, it is important to know the optimal parameters: pH, texture, structure, type and quantity of microorganisms, temperature and extraction time, used in the extraction process. At the same time, it can be said that there is still a number of controversies worldwide, especially in terms of the effectiveness of the process (i.e., the optimal intervention time needed to obtain a satisfactory degree of depollution) which vary according to the specific characteristics of the soil and the initial concentration of the pollutant and the type of pollutant. It should also be mentioned that, due to the high degree of inhomogeneity of the soils, it is particularly important to accumulate as many experimental results as possible. Accumulating the necessary data generates the possibility to assess, on a case-by-case basis, the parameters necessary to obtain a satisfactory degree of decontamination, such as the duration of intervention, the concentration and quantity of the pollutant, etc.

The aim of the paper is to highlight the factors that influence the yield of the bioleaching process, these being mainly the amount of bioleaching solution and the duration of the extraction process of potentially toxic elements (metals), in relation to the environmental risk assessment. The use of yield–time correlation equations allows the identification of the optimal exposure time on the bioleaching extraction’s potentially toxic elements to obtain optimal results.

## 2. Materials and Methods

### 2.1. Soil Sampling

In order to study the assesed parameters, soil samples from two different areas in Romania were extracted. These areas are particulary known for generating industrial activities (area P1—Maramureș County and area P2—Alba County). Soil from three different depths was analyzed for each of the two areas: 0–10 cm, 10–20 cm and 20–30 cm. The analysis of the data was performed according to the Romanian standard STAS 7184/1-84 and were processed according to ISO 11464:1998 [52,53]. The investigated areas are specific to mining areas and adjacent to these types of activities, being known as polluted areas in Romania [54,55,56].

### 2.2. Soil Analysis

Characterization of the soil samples was carried out in terms of pH, texture, structure and content of metals (Cu, Pb, Cr and Ni). Soil pH was determined in soil/water extract 1/5 (*w*/*v*) using a HANNA pH meter. Soil texture was determined by gravimetric method with RETSCH AS 200 sieve and soil structure was determined by Sekera method. Soil metal content was determined by atomic absorption spectrometry (AAS) with a SHIMADZU AA-6800 spectrometer (Shimadzu, Tokyo, Japan) using the aqua regia digestion. The soil samples were dried, grounded to a fine powder and sieved through a 100 μm sieve. Three grams of each sample of soil were weighed into a beaker and 7 mL of HCl and 21 mL of HNO_3_ were added. The mixture was then refluxed for 2 h. After cooling to room temperature, the supernatant was filtered and diluted to 100 mL.

### 2.3. Bioleaching Process

The extraction of metals from contaminated soil was achieved by bioleaching using 180 *Thiobacillus ferrooxidans* (TF) microorganisms inoculated in 9K medium in different quantities: 20 mL and 40 mL. The 9K medium contained: (NH_4_)2SO_4_—3.0 g; KCl—0.1 g; K_2_HPO_4_—0.5 g; MgSO_4_·7H_2_O—0.5 g; Ca(NO_3_)_2_·4H_2_O—0.01 g; FeSO_4_·7H_2_O—44.2 g; and distilled water up to 1000 mL [57].

From each soil sample, 10 g of soil (from the 2 mm soil fraction) was weighed and 20 mL and 40 mL of 9K medium was added. Samples were stirred using an orbitally oscillating–rotating platform shaker (200 rpm) for 2 h, 4 h, 6 h, 8 h, 10 h and 12 h. Research was carried out in laboratory conditions under constant temperature, real air humidity and ventilation conditions (T = (27 ± 1) °C, RH = (65 ± 2)%), without forced ventilation of ambient air. At regular time intervals (2 h, 4 h, 6 h, 8 h, 10 h, 12 h) the leachate was filtered and the concentrations of the four metals (Cu, Pb, Cr, Ni) were determined by Atomic Absorption Spectrometry (AAS). To ensure repeatability and reproducibility, the results were recorded as the average of three successive measurements.

### 2.4. Ecological Risk Assessment Methodology

Based on previous studies and results [8,27,28,58,59,60,61], C_f_ contamination index and E_r_ ecological risk parameters were calculated for each sample and for each type of pollutant and measurable indices for general assessment of the soil condition were analyzed, i.e., C_d_, PLI and PERI.

The calculation of the parameters were performed using background values of: Cu: 25; Pb: 20; Cr: 35; Ni: 20 [62]. The degree of C_d_ contamination was calculated by summing the contamination indices according to Equation (8):

(8) $$C_{f}=\frac{C_{\mathrm{Ai}}}{C_{\mathrm{Ni}}}-1$$

where,

C_Ai_—analyte concentration,
C_Ni_—background value (in the case of soil).

(9) $$C_{d}=\sum_{i=1}^{n}\left(C_{f}\right)$$

(10) $$\mathrm{PLI}=\sqrt[{n}]{c_{f1}{*c}_{f2}{*c}_{f3}*\ldots .{*c}_{\mathrm{fn}}}$$

(11) $$\mathrm{Er}=C_{f}{*T}_{r}$$

where,

E_r_—ecological risk index of the metal i,
C_f_—contamination factor of the metal i,
T_r_—metal toxicity response coefficient for each metal: Cu: 5, Pb: 5, Cr: 2, Ni: 5 [8,63].

(12) $$\mathrm{PERI}=\sum_{i=1}^{n}\mathrm{Er}$$

The results obtained from the calculations are interpreted according to the values of the corresponding parameters by comparison with the information available in the literature (Table 1) and presented also in colors, in order to easily asses the degree of pollution and/or ecological risk.

During the entire experimental program, the evolution of the “soil health status” was monitored. This parameter was expressed as the accumulation of all the factors influencing this aspect, as well as the kinetics of the evolution of each type of pollutant in relation to the duration of exposure to the bioleaching solution, the amount of 9K medium used, the depth of soil sampling and the pH of the soil. The kinetics of the decontamination process was followed by decreasing the concentration of the pollutant in relation to the time of action of each type of solution.

The effectiveness of the extraction process (ER) was determined with Equation (13) [64]:

(13) $$\mathrm{removal}\;\mathrm{efficiency}\;(\%)=\frac{C_{i}-{\;C}_{f}\;}{C_{i}}\times 100$$

where:

C_i_ is the initial metals’ concentration (mg kg^−1^) of soil;
C_f_ is the final concentration of metals (mg kg^−1^) in soil, after soil bioleaching treatment.

In order to estimate the time needed to apply the process until a satisfactory degree of depollution is obtained, a series of equations correlating the yield with the time of action were identified on the basis of experimental data. In this respect, the identified correlation relationship was considered acceptable if the condition R^2^ ≥ 0.9 was satisfied.

## 3. Results and Discussions

### 3.1. Analysis and Assessment of Ecological Risks for Initial Soil Samples

The results of the pH tests indicate that all soil samples show a low pH (acid reaction) with varying degrees of acidity. Samples P1 show a pH value of 5.2–5.5 (medium acidic reaction), while samples P2 show a pH value of 2.3–2.5 (highly acidic reaction). In both investigated locations, the soil was found to be well structured and has a sandy loam texture with the following composition: 21.8% clay, 40.2% silt and 38% sand, according to the USDA classification [65].

**Figure 2:**
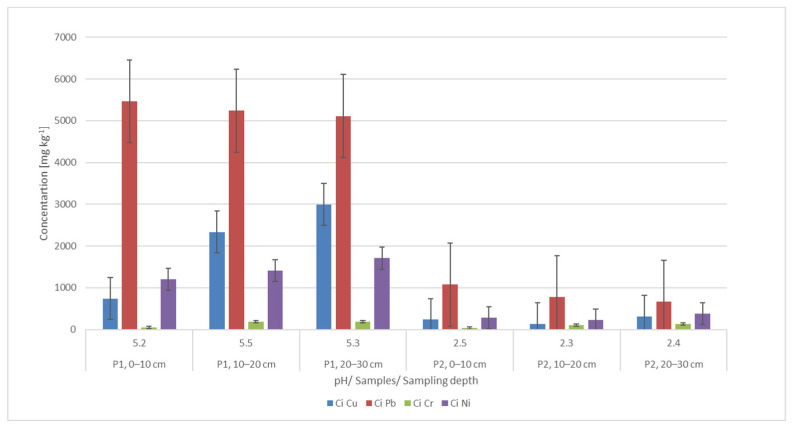
Initial metal concentration and pH of soil samples as a function of sampling location and depth.

Initial testing of the soil samples indicated that, depending on the location and depth of extraction, metal concentrations vary substantially. As shown in Figure 2, a slight decrease in Pb concentration with an increasing depth of investigation was observed at both investigated locations. On the other hand, the Cu, Ni and Cr content increased with the depth of investigation. This can be explained by the fact that Pb is associated with the acid-soluble and reducible phases, while the other metals studied are mainly associated with the oxidization phase. These metal migration trends in the top layer (up to 30 cm) are in agreement with the literature. Regarding the different concentrations of metals, between the two areas investigated, it can be appreciated that the fact that these areas are part of two different regions, with two different types of exploitation and pollution, lead to the presented results.

**Figure 3:**
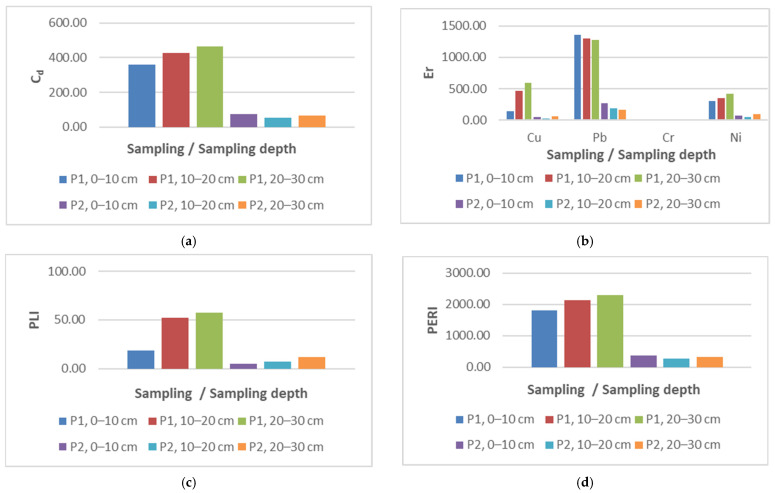
Parameters calculated for the initial concentration: (**a**) C_d_—degree of contamination; (**b**) E_r_—environmental risk index; (**c**) PLI—pollution loading index; and (**d**) PERI—potential environmental risk index.

The metal concentration values of the investigated samples show that they exceed the normal values: 20 mg kg^−1^ (Cu, Pb and Ni) and 30 mg kg^−1^ (Cr), according to the Romanian legislation (Order no. 756/1997). In the case of sample P1, the concentrations are so high that they even exceed the action threshold: 1000 mg kg^−1^ (Pb), 500 mg kg^−1^ (Cu and Ni) [66]. Comparing the calculated indicators, C_d_, E_r_, PLI and PERI with the admissibility thresholds (Figure 3), it can be seen that all these indicators indicate the need for decontamination intervention. The sources of the pollution in this area are the mining activities carried out in the area over the years. At the moment, all the mines in the area are closed.

### 3.2. Environmental Risk Assessment during the Bioleaching Process

**Figure 4:**
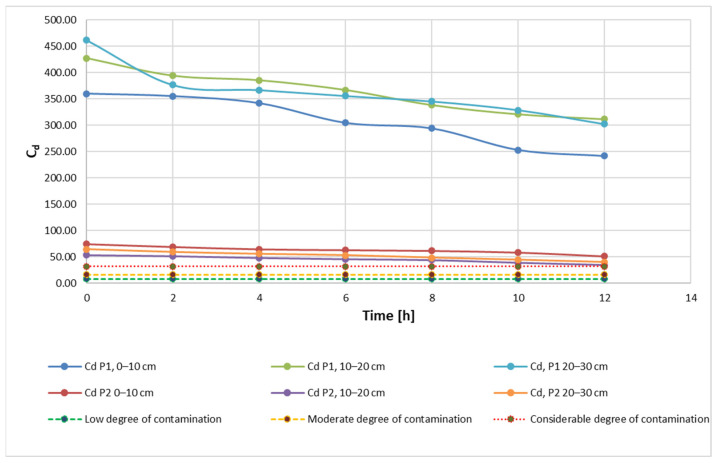
C_d_—degree of contamination during the extraction process according to admissibility limits and sampling depth using 20 mL 9K medium.

After applying the metal extraction treatment by bioleaching, with 20 mL and 40 mL of 9K medium, the results obtained show that the C_d_ indicator decreases steadily over time (Figure 4), indicating the overall efficiency of the decontamination process. From the shape of the curves, it can be seen that the variation of this indicator is directly influenced by the duration of the soil being exposed to the bioleaching solution, i.e., a linear dependence of the C_d_ parameter on time.

On the other hand, soil with a more acidic pH, but also with an initially lower contaminant content, shows a variation much closer to the ideal situation of linear variation. However, the plot area indicates a slower rate of change of the C_d_ indicator in this case (compared to the more contaminant-rich soil with a higher pH). These observations suggest that, in addition to the length of time of exposure to the soil decontamination method, the evolution of the C_d_ indicator is influenced by both the concentration of contaminant metals and soil pH. The positioning of the experimental results, grouped for each soil type, even for the three different sampling depths, may be an indication that the sampling depth is not a factor directly influencing the evolution of the extraction process.

**Figure 5:**
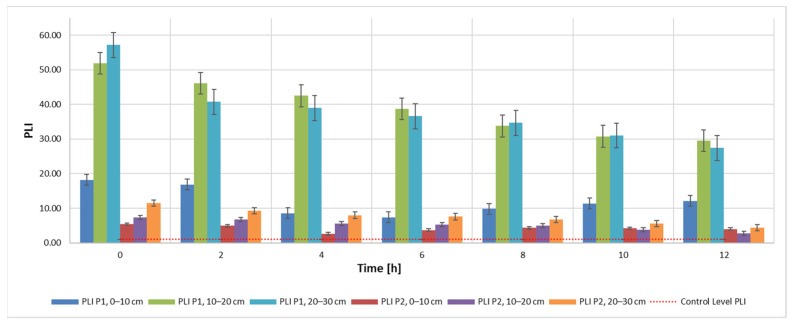
PLI—pollution load index during the extraction process according to admissibility limits and sampling depth using 20 mL 9K medium.

**Figure 6:**
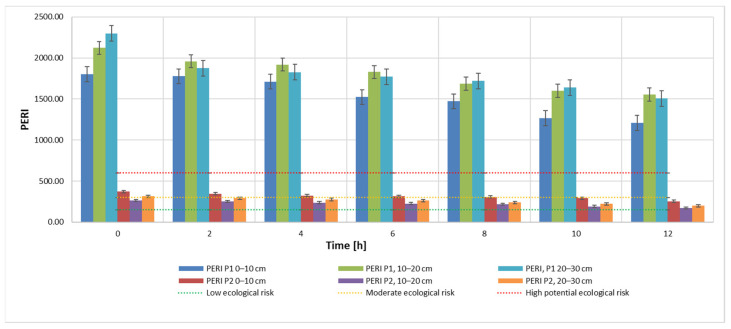
PERI—potential ecological risk index during the extraction process according to admissibility limits and sampling depth using 20 mL 9K medium.

Results obtained for the C_d_ indicator are also found for the other two indicators, PLI (Figure 5) and PERI (Figure 6). The evolution of these indicators, on the other hand, is influenced by the evolution of the pollutant concentration which is a direct influence in the evolution of the E_r_ indicator.

As shown in the figures above, in contradiction to the evolution of the concentrations of the three polyunsaturated metals, Cu, Pb and Ni, the Cr concentration shows a different variation. During the first 4–5 h of treatment, the concentration and its impact on the general soil characterization decreases. Afterwards, a continuous and constant increase of this parameter in terms of pollution is observed.

**Figure 7:**
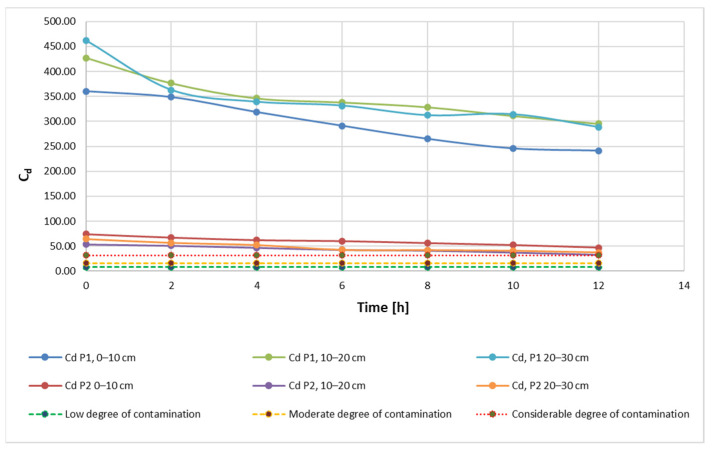
C_d_—degree of contamination during the extraction process according to the admissibility limits and sampling depth using 40 mL 9K medium.

**Figure 8:**
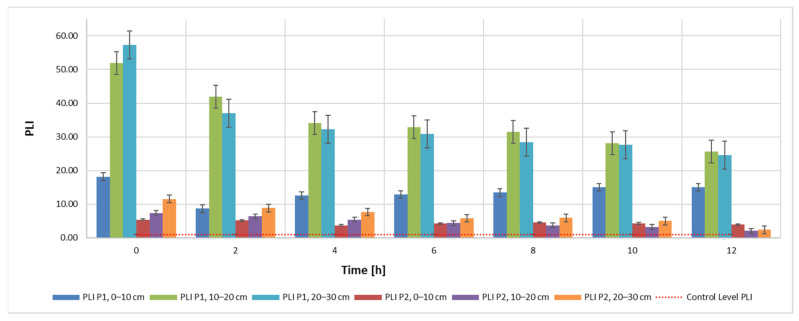
PLI—pollution load index during the extraction process according to admissibility limits and sampling depth using 40 mL 9K medium.

**Figure 9:**
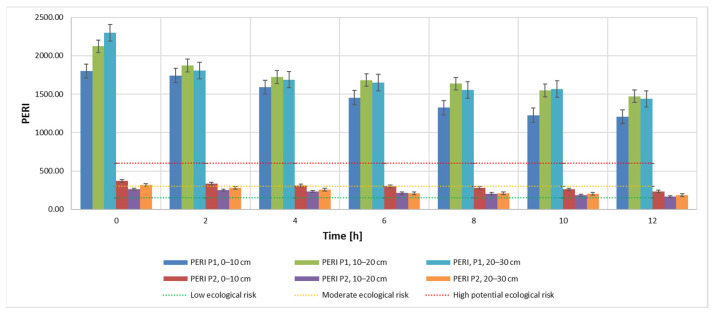
PERI—potential ecological risk index during the extraction process depending on the admissibility limits and sampling depth using 40 mL 9K medium.

This can be explained by the specificity of the bioleaching process using TF microorganisms, whose metabolism contributes to the transition from Cr(IV) to Cr(III) [6,11,67]. Bacteria can degrade Cr(VI) to Cr(III) in anaerobic environments, where it uses chromate as a terminal electron acceptor, and in aerobic environments via cellular depleting agents. The transformation of Cr(VI) to Cr(III) leads to a reduction of toxicity, showing the stabilization of Cr in the soil with little migration of Cr to the plant [6]. It can also be noticed that, although the measuring equipment records this Cr concentration, the advantage of the process is the ability of the element to change from its form which is hazardous to human health to a less hazardous form. In accordance with the specifications in the literature, results show a very high degree of contamination (Cd > 32), a pollution index PLI > 1 and a significant ecological risk for both soil samples that were analyzed in this study (Figure 7, Figure 8 and Figure 9). Results obtained on both soil samples show that interventions are required for the decontamination and reduction of both the pollution index and the environmental risk. Although the extraction process by bioleaching was applied for 12 h, the aforementioned parameters, at the end of the test, were not positioned in safe zones, which indicates the need for further treatment.

Most of the soil samples presented C_f_ ≥ six values, resulting in a very high degree of pollution (Table 2). Although samples have shown high values in terms of the analyzed parameters, the C_r_ parameter showed values < one for most samples. A relatively small number of samples (seven samples) had a C_f_ ranging from three to six, indicating considerable contamination (Cr = Ni < Cu). The C_f_ contamination index has the following order: Pb > Cu > Ni > Cr. The soil in the studied areas shows a high degree of contamination, with the PLI pollution index greater than one and the C_d_ contamination degree greater than 32 at the end of the 12 h of treatment, regardless of the amount of 9K medium used.

The E_r_ ecological risk factor values for the studied areas ranged from 0.06 to 1361.50 (Table 3). Samples from the investigation area P1 (ER > 320) show very high values in all studied cases (Pb, Cu and Ni). For samples of P2 (ER < 40), an increase in Pb is observed, which highlights an increased risk or considerable risk of contamination. P2 samples generally show a low or moderate environmental risk for Cu, Cr and Ni. Cr is the only element with a low environmental risk regardless of the location of the soil sample. Analyzing the degree of ecological risk for the samples studied, the following order can be established: Pb > Ni > Cu > Cr.

**Figure 10:**
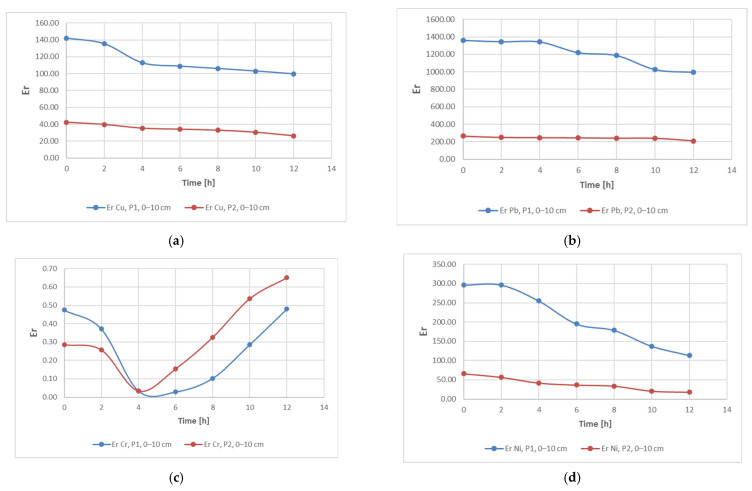
Evolution of the E_r_ indicator as a function of metal, location and sampling depth: (**a**) Cu, (**b**) Pb, (**c**) Cr and (**d**) Ni.

Figure 10 shows the evolution of the E_r_ parameter for samples P1 and P2 (extracted from a 0–10 cm depth). As seen in the figure, the samples have similar evolutions in terms of the analyzed parameters.

The values obtained for the potential environmental risk index (PERI) for samples extracted from area P1 range from 1204.08 to 2297.85, far exceeding the PERI = 600 value. This indicates a significantly high degree. The PERI values obtained for samples extracted from area P2 indicate a moderate ecological risk, except two initial samples (depth 0–10 cm and 20–30 cm, respectively).

The C_d_ contamination degree, and the PLI pollution parameters have very high values for all 18 samples investigated. The PERI indicator shows high concentrations for samples extracted from area P1 and a moderate ecological risk for those from area P2. The results of the analyzed indicators suggest that the area has a high level of soil pollution and indicate a strong anthropogenic influence on the soil in the studied area.

**Figure 11:**
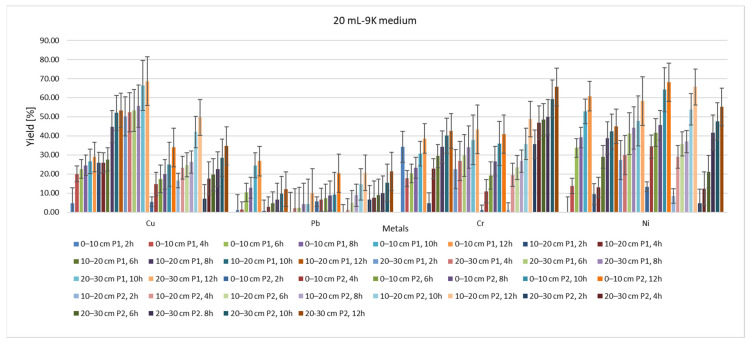
Yield of extraction process with 20 mL of 9K medium.

**Figure 12:**
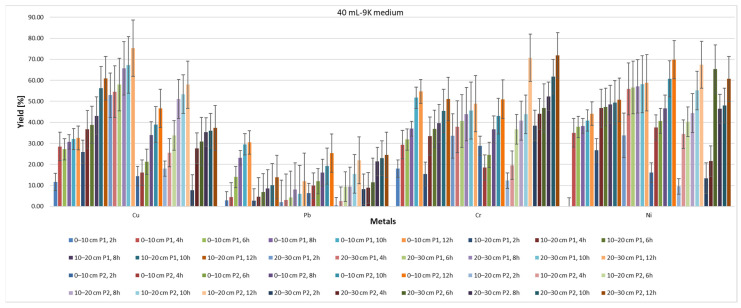
Yield of extraction process with 40 mL of 9K medium.

**Figure 13:**
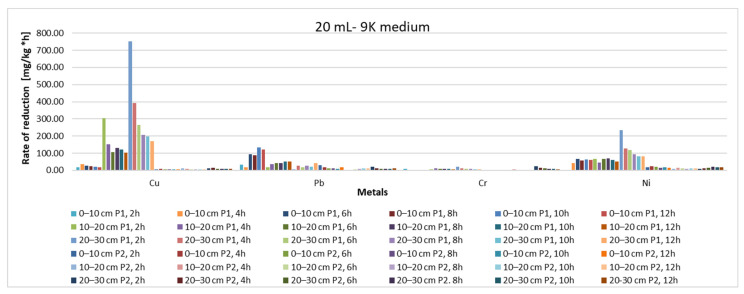
Reduction rate of pollutant concentration, depending on sample, sampling depth, amount of 9K medium and extraction time (20 mL 9K medium).

**Figure 14:**
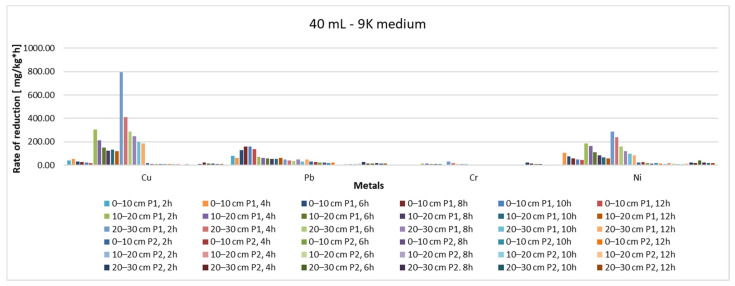
Reduction rate of pollutant concentration, depending on sample, sampling depth, amount of 9K medium and extraction time (40 mL 9K medium).

From the point of view of the yield of the extraction process (Figure 11 and Figure 12), it can be said that, regardless of whether 20 mL or 40 mL 9K medium was used, the depth of the sample extraction does not directly influence this parameter or the rate of reduction of the pollutant concentration (Figure 13 and Figure 14). Indirectly, through the concentration of the pollutant in the analyzed samples, both the yield and the speed of the pollutant concentration reduction process are influenced, but a direct correlation, expressed by a mathematical dependence function of each of the two measurable indicators with the depth of extraction of the soil samples, cannot be made.

On the other hand, it can be seen that the type of pollutant directly influences both the speed of the extraction process and its rate of reduction from the soil. Thus, in the case of Pb, the lowest yield is observed for the use of 20 mL of 9K medium, for a soil with a pH of 5.2–5.5. In contrast, in the case of soil with a pH of 2.3–2.5, the lowest yield of the depollution process is observed for Cr. In the two studied areas, the depollution process has a good yield in the case of Cu. In terms of the rate at which the pollutant concentration is reduced, in the case of soil with pH 5, poor results are observed in the case of Cr. All these observations lead to the appreciation that, in addition to the type of pollutant, the duration of action and the amount of 9K solution used, the initial concentration of the pollutant is of great importance in the mechanism of depollution by this process. A high concentration of pollutant will determine a good yield of the depollution process as well as a good speed of reduction of the concentration of the pollutant. This, however, varies greatly depending on the type of metal pollutant. The results obtained for Pb (10–32%) and Ni (44–68%) using TF are higher compared to the yields obtained using *Aspergillus niger*: Pb: 13% and Ni: 21% [68]. The same can be observed for Cu (39–72%), and when using *Acidithiobacillus*, *Sulfobacillus* and *Ferroplasma*, where Cu amounts to 27% [69], *Pleurotus florida* (18%) and *Pseudomonas* spp. (16.6%) [70].

**Figure 15:**
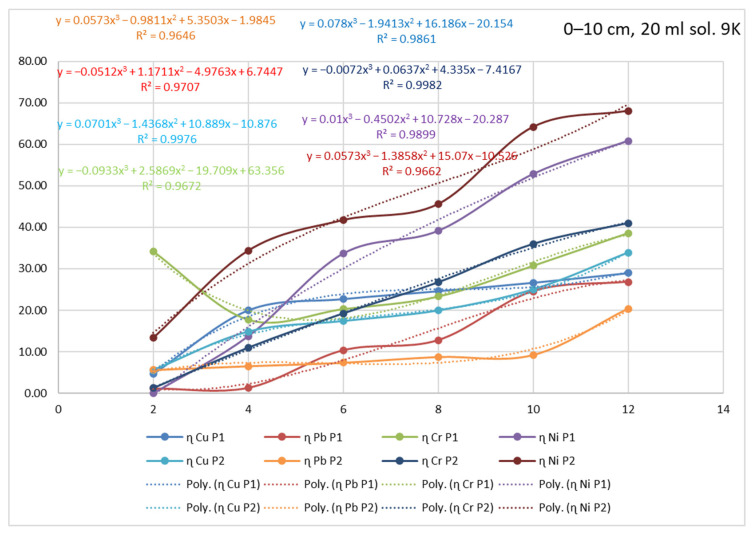
Identification of equations and yield–time correlation indices for P1 and P2 samples taken from 0–10 cm depth and treated with 20 mL of 9K medium.

By analyzing the yield of the extraction process for each type of metal, in relation to the time of action of the 9K medium, and by analyzing the plot area, it can be seen that this process indicator could be modeled based on polynomial equations. Figure 15 shows the identified equations and yield–time correlation indices for the situation of P1 and P2 samples extracted from a 0–10 cm depth and treated with 20 mL of 9K medium. The results indicate that the yield of the extraction process varies, for all the analyzed metals, with respect to time according to polynomial 3-degree equations. Therefore, the evolution of the extraction process could be assessed, in time, with significantly good accuracy (correlation index R^2^ > 0.95) for each type of polluted metal.

Table 4 shows the correlation equations of the extraction process yield with time and the correlation factor R^2^. However, the fact that the mathematical equations identified are of degree three (ax^3^ + bx^2^ + cx + d) leads to the hypothesis that, although time might be the main determinant of the yield process, there are other influencing factors. Among them, the most important is the type of polluting metal and its concentration. The importance of identifying these yield–time correlation equations lines is the possibility of demonstrating the influence that the exposure time has on the yield of the process, or more precisely, the need to carry out longer tests to demonstrate and quantify the efficiency of this method of depollution. On the other hand, based on these equations, it is possible to estimate the duration necessary for the process of extraction by bioleaching to obtain the best results, i.e., maximum yield, and to restore the ecological balance of the soil. Analyzing the relationships, two exceptions are observed for Pb (R^2^ = 0.8697) and Ni (R^2^ = 0.734). These equations are not considered to be representative for assessing the evolution of the decontamination phenomenon. It is considered that these situations may be due to the high degree of inhomogeneity of the soil sample.

When analyzing the rate of reduction of the pollutant concentration, different results can be observed, as the kinetics of this process can be appreciated, over time, with a significantly good accuracy (R^2^ >0.95) for some polluted metals, respectively, with a lower accuracy for other samples analyzed (0.85 < R^2^ < 0.9). The results show that for samples extracted from area P1 (in terms of Pb) and area P2 (in terms of Cu) the kinetics of the phenomenon cannot be appreciated as having an evolution characterized by an equation as a function of time. Therefore, the rate of depollution is considered to depend on a combination of several factors, not just time.

The results obtained in the research regarding extraction yields for Pb (10–32%) support the claims of Blais J.F. (10–54%) [43], Chen S.Y. (16–60%) [50] and Beolchini F. (18%) [40], but the yields are slightly lower than those obtained by Chen S.Y. (33–72%) [33], Zhou Q. (39%) [42] and Li Q. (75–84%) [49].

The results obtained in the case of Cu and Pb are similar to those obtained by other researchers, while in the case of Cr and Ni, higher yields were obtained than in most of the studies studied. For example, Cr was extracted by 39–72%, while Beolchini F. (14%) [40], Blais J.F. (10–41%) [43] and Chen S.Y (9–20%) [51] obtained lower yields, and at the opposite pole are the results obtained by Li Q. who extracted 53–92% of Cr [49].

The Ni concentrations extracted (44–68%) are comparable to those of Chen S.Y. (35–65%) [51], much lower than those of Blais J.F. (69–92%) [43], Beolchini F. (78%) [40], Kamizela T. (90%) [33], but higher than the yields obtained by Chen S.Y. (10–47%) [51].

The Cu yield (39–72%) falls within the same values as those obtained by Duyusen G. 44% [39] and Beolchini F. 46% [40], but most studies in the literature show higher yields with values above 80% [31,43,47,48] or even above 95% [44,45,49,50].

The investigated samples show very high values for the investigated indices (C_d_, PLI and PERI) indicating that the investigated areas have a high level of soil pollution with a strong anthropogenic influence. This demonstrates that the two areas show complex, multi-element contamination, typical of areas with a long history of mining-specific industrial activities, and is comparable with the results of studies reported by other researchers [71,72,73].

## 4. Conclusions

Results obtained on the analyzed soil samples used in the current study showed that the soil is well structured, has a sandy loam texture, a medium acidic to highly acidic reaction and is highly contaminated with metals, exceeding normal values or even exceeding the alert and intervention thresholds, according to Romanian legislation.

Analyzing the ecological risk (Er) (Pb > Ni> Cu > Cr) and the contamination index (C_f_) (Pb > Cu > Ni > Cr) revealed that the greatest danger is posed by Pb, and at the opposite pole is Cr. Analyzing the PERI, we can conclude that the potential ecological risk index differs in the two studied areas, so that the samples from area P1 predict a significantly high risk and those from area P2, a moderate ecological risk. Thus, after analyzing the results of C_d_, PLI and PERI, which have very high values for all the samples analyzed, it can be stated that the soils in the studied area have a very high degree of pollution, which is caused by anthropogenic activity in the area.

The results obtained by the extraction of metals by bioleaching allowed us to highlight the factors influencing the yield of the process, these being mainly the amount of bioleaching solution and the duration of the extraction process. It was found, for all metals investigated, that a higher amount of bioleaching solution allows for the obtaining of a higher metal extraction efficiency. It can be appreciated that a longer time duration leads to a higher yield of the extraction process.

The identification of the yield–time correlation equations allows the possibility of demonstrating the influence that the exposure time has on the yield of the process and the appreciation of the necessary duration of the extraction process by bioleaching to obtain the best results, i.e., maximum yield, and to restore the ecological balance at soil level.

## Figures and Tables

**Table 1:** Metal pollution indices used for soil quality assessment.

**PARAMETERS**	**C_f_—Contamination factor** [8,27,28]		C_f_ < 1—Low contamination factor
			1 ≤ C_f_ < 3—Moderate contamination factor
			3 ≤ C_f_ < 6—Considerable contamination factor
			C_f_ ≥ 6—Very high contamination factor
	**C_d_**—**Degree of Contamination** [8,58]		C_d_ < 8—Low degree of contamination
			8 ≤ C_d_ ≤ 16—Moderate degree of contamination
			16 ≤ C_d_ ≤ 32—Considerable degree of contamination
			C_d_ > 32—Very high degree of contamination
	**PLI—Pollution Load Index** [27,28,59]		PLI < 1—Not polluted soil
			PLI = 1—Soil with normal background level
			PLI > 1—Polluted soil
	**Er—Ecological risk index** [8,60]		E_r_ < 40—Low ecological risk
			40 < E_r_ ≤ 80—Moderate ecological risk
			80 < E_r_ ≤ 160—Considerable ecological risk
			160 < E_r_ ≤ 320—High ecological risk
			E_r_ > 320—Serious ecological risk
	**PERI—Potential Ecological Risk Index** [8,61]		PERI < 150—Low ecological risk
			150 ≤ PERI < 300—Moderate ecological risk
			300 ≤ PERI < 600—High potential ecological risk
			PERI ≥ 600—Significantly high ecological risk

**Table 2:** Calculated values for the C_f_ (contamination factor), C_d_ (contamination degree) and PLI (pollution load index).

Sample				C_f_				C_d_	PLI
Code	Sampling Depth [cm]	Time [h]	9K Medium [mL]	Cu	Pb	Cr	Ni		
**P1**	0–10	Initial	-	28.44	272.30	0.24	59.30	360.28	18.17
		Final (after 12 h)	20	19.92	198.95	0.24	22.65	241.76	12.12
			40	18.84	189.10	0.44	32.70	241.08	15.05
	10–20	Initial	-	92.44	261.15	4.34	69.35	427.28	51.93
		Final (after 12 h)	20	42.60	229.60	2.07	37.70	311.97	29.56
			40	35.52	224.60	1.61	33.70	295.43	25.67
	20–30	Initial	-	118.84	254.65	4.20	84.40	462.09	57.23
		Final (after 12 h)	20	36.60	229.00	1.94	34.65	302.19	27.41
			40	28.60	224.00	1.66	34.15	288.41	24.54
**P2**	0–10	Initial	-	8.44	52.75	0.14	13.25	74.58	5.39
		Final (after 12 h)	20	5.24	41.80	0.33	3.55	50.92	3.99
			40	4.04	39.10	0.44	3.30	46.88	3.89
	10–20	Initial	-	4.32	37.55	1.80	10.05	53.72	7.36
		Final (after 12 h)	20	1.68	29.60	0.43	2.80	34.51	2.79
			40	1.24	29.10	0.18	2.60	33.12	2.03
	20–30	Initial	-	11.44	32.25	2.66	18.05	64.40	11.53
		Final (after 12 h)	20	7.12	25.15	0.26	7.55	40.08	4.32
			40	6.80	24.10	0.03	6.50	37.43	2.35
Legend:	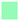 C_f_ < 1—Low contamination factor 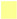 C_f_: 1–3—Moderate contamination factor 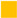 C_f_: 3–6—Considerable contamination factor				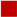 C_f_ ≥ 6—Very high contamination factor 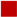 C_d_ > 32—Very high degree of contamination 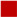 PLI > 1—Polluted soil				

**Table 3:** Ecological risk index E_r_ and PERI potential ecological risk, recorded after 12 h of treatment with 20 mL and 40 mL 9K medium solution, respectively.

Sample				E_r_				PERI
Code	Sampling Depth [cm]	Time [h]	9K Medium [mL]	Cu	Pb	Cr	Ni	
**P1**	0–10	Initial	-	142.20	1361.50	0.47	296.50	1800.67
		Final (after 12 h)	20	99.60	994.75	0.48	113.25	1208.08
			40	94.20	945.50	0.88	163.50	1204.08
	10–20	Initial	-	462.20	1305.75	8.69	346.75	2123.39
		Final (after 12 h)	20	213.00	1148.00	4.14	188.50	1553.64
			40	177.60	1123.00	3.23	168.50	1472.33
	20–30	Initial	-	594.20	1273.25	8.40	422.00	2297.85
		Final (after 12 h)	20	183.00	1145.00	3.89	173.25	1505.14
			40	143.00	1120.00	3.31	170.75	1437.06
**P2**	0–10	Initial	-	42.20	263.75	0.29	66.25	372.49
		Final (after 12 h)	20	26.20	209.00	0.65	17.75	253.60
			40	20.20	195.50	0.88	16.50	233.08
	10–20	Initial	-	21.60	187.75	3.60	50.25	263.20
		Final (after 12 h)	20	8.40	148.00	0.87	14.00	171.27
			40	6.20	145.50	0.36	13.00	165.06
	20–30	Initial	-	57.20	161.25	5.31	90.25	314.01
		Final (after 12 h)	20	35.60	125.75	0.51	37.75	199.61
			40	34.00	120.50	0.06	32.50	187.06
Legend:	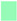 E_r_ < 40—Low ecological risk 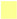 E_r_: 40–80—Moderate ecological risk 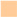 E_r_: 80–160—Considerable ecological risk 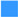 E_r_: 160–320—High ecological risk 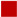 E_r_ > 320—Serious ecological risk				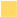 PERI: 150–300—Moderate ecological risk 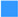 PERI: 300–600—High potential ecological risk 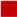 PERI ≥ 600—Significantly high ecological risk			

**Table 4:** Correlation equations of extractive process yield with time and correlation factor R^2^.

Code	Sampling Depth [cm]	Pollutant	Correlation Equation of Extraction Yield with Time	Correlation Index, R^2^
**20 mL 9K medium**				
P1	0–10	**Cu**	y = 0.078x^3^ − 1.9413x^2^ + 16.186x − 20.154	R^2^ = 0.9861
		**Pb**	y = −0.0512x^3^ + 1.1711x^2^ − 4.9763x + 6.7447	R^2^ = 0.9707
		**Cr**	y = −0.0933x^3^ + 2.5869x^2^ − 19.709x + 63.356	R^2^ = 0.9672
		**Ni**	y = 0.01x^3^ − 0.4502x^2^ + 10.728x − 20.287	R^2^ = 0.9899
	10–20	**Cu**	y = −0.1323x^3^ + 2.9061x^2^ − 15.246x + 46.49	R^2^ = 0.9751
		**Pb**	y = −0.0002x^3^ + 0.0314x^2^ + 0.7325x − 0.8138	R^2^ = 0.9982
		**Cr**	y = 0.0557x^3^ − 1.5327x^2^ + 15.658x − 20.428	R^2^ = 0.9927
		**Ni**	y = −0.0779x^3^ + 1.3926x^2^ − 2.5333x + 8.6472	R^2^ = 0.9811
	20–30	**Cu**	y = −0.0195x^3^ + 0.5892x^2^ − 3.0349x + 54.717	R^2^ = 0.9442
		**Pb**	y = 0.0309x^3^ − 0.5623x^2^ + 3.5127x − 4.8439	R^2^ = 0.9667
		**Cr**	y = 0.0114x^3^ − 0.1962x^2^ + 2.8585x + 17.582	R^2^ = 0.9985
		**Ni**	y = 0.0204x^3^ − 0.3889x^2^ + 5.0498x + 17.721	R^2^ = 0.9679
P2	0–10	**Cu**	y = 0.0701x^3^ − 1.4368x^2^ + 10.889x − 10.876	R^2^ = 0.9976
		**Pb**	y = 0.0573x^3^ − 0.9811x^2^ + 5.3503x − 1.9845	R^2^ = 0.9646
		**Cr**	y = −0.0072x^3^ + 0.0637x^2^ + 4.335x − 7.4167	R^2^ = 0.9982
		**Ni**	y = 0.0573x^3^ − 1.3858x^2^ + 15.07x − 10.526	R^2^ = 0.9662
	10–20	**Cu**	y = 0.0322x^3^ − 0.4043x^2^ + 3.4665x + 11.779	R^2^ = 0.9548
		**Pb**	y = −0.0095x^3^ + 0.3439x^2^ − 1.1457x + 1.1673	R^2^ = 0.9989
		**Cr**	y = 0.129x^3^ − 2.7375x^2^ + 20.897x − 30.102	R^2^ = 0.9934
		**Ni**	y = 0.122x^3^ − 2.5767x^2^ + 20.829x − 22.926	R^2^ = 0.9794
	20–30	**Cu**	y = 0.0581x^3^ − 1.245x^2^ + 10.208x − 8.3601	R^2^ = 0.9869
		**Pb**	y = 0.019x^3^ − 0.2151x^2^ + 1.2339x + 4.9123	R^2^ = 0.9924
		**Cr**	y = 0.0645x^3^ − 1.3256x^2^ + 10.484x + 20.104	R^2^ = 0.9706
		**Ni**	y = −0.0881x^3^ + 1.7938x^2^ − 4.9839x + 8.3115	R^2^ = 0.9875
**40 mL 9K medium**				
P1	0–10	**Cu**	y = 0.0761x^3^ − 1.9138x^2^ + 15.763x − 11.866	R^2^ = 0.9259
		**Pb**	y = −0.0866x^3^ + 1.7478x^2^ − 6.8215x + 10.062	R^2^ = 0.9982
		**Cr**	y = 0.0075x^3^ − 0.1242x^2^ + 4.1427x + 11.239	R^2^ = 0.9583
		**Ni**	y = 0.2053x^3^ − 5.0242x^2^ + 39.427x − 59.204	R^2^ = 0.9726
	10–20	**Cu**	y = 0.0237x^3^ − 0.4359x^2^ + 5.534x + 17.352	R^2^ = 0.9646
		**Pb**	y = 0.0128x^3^ − 0.2366x^2^ + 2.2303x − 1.0236	R^2^ = 0.9965
		**Cr**	y = 0.0966x^3^ − 2.2616x^2^ + 18.62x − 12.941	R^2^ = 0.986
		**Ni**	y = 0.1115x^3^ − 2.7581x^2^ + 21.812x − 6.0412	R^2^ = 0.9615
	20–30	**Cu**	y = −0.0089x^3^ + 0.2983x^2^ − 0.4406x + 52.492	R^2^ = 0.9773
		**Pb**	y = 0.0165x^3^ − 0.2933x^2^ + 2.2293x − 1.6559	R^2^ = 0.8697
		**Cr**	y = 0.0114x^3^ − 0.2845x^2^ + 3.5453x + 27.473	R^2^ = 0.9972
		**Ni**	y = 0.1245x^3^ − 3.0828x^2^ + 24.233x − 2.42	R^2^ = 0.9588
P2	0–10	**Cu**	y = −0.0579x^3^ + 1.3477x^2^ − 5.7293x + 20.904	R^2^ = 0.9894
		**Pb**	y = 0.0168x^3^ − 0.2763x^2^ + 2.8752x + 1.5814	R^2^ = 0.9967
		**Cr**	y = −0.1264x^3^ + 3.0672x^2^ − 18.985x + 54.917	R^2^ = 0.982
		**Ni**	y = 0.0954x^3^ − 2.084x^2^ + 18.132x − 11.345	R^2^ = 0.9766
	10–20	**Cu**	y = −0.0748x^3^ + 1.3971x^2^ − 2.7604x + 18.546	R^2^ = 0.9819
		**Pb**	y = 0.018x^3^ − 0.288x^2^ + 3.0948x − 4.9287	R^2^ = 0.9718
		**Cr**	y = 0.1227x^3^ − 2.3933x^2^ + 18.265x − 17.483	R^2^ = 0.9607
		**Ni**	y = 0.1477x^3^ − 3.2954x^2^ + 26.529x − 30.769	R^2^ = 0.9905
	20–30	**Cu**	y = 0.0826x^3^ − 2.1972x^2^ + 19.483x − 22.508	R^2^ = 0.9825
		**Pb**	y = −0.0656x^3^ + 1.3786x^2^ − 6.4111x + 16.04	R^2^ = 0.9709
		**Cr**	y = 0.0245x^3^ − 0.2959x^2^ + 3.2722x + 33.177	R^2^ = 0.9967
		**Ni**	y = 0.1461x^3^ − 3.7235x^2^ + 31.939x − 41.032	R^2^ = 0.734

## Data Availability

Not applicable.
